# Imipramine Is an Orally Active Drug against Both Antimony Sensitive and Resistant *Leishmania donovani* Clinical Isolates in Experimental Infection

**DOI:** 10.1371/journal.pntd.0001987

**Published:** 2012-12-27

**Authors:** Sandip Mukherjee, Budhaditya Mukherjee, Rupkatha Mukhopadhyay, Kshudiram Naskar, Shyam Sundar, Jean Claude Dujardin, Anjan Kumar Das, Syamal Roy

**Affiliations:** 1 CSIR-Indian Institute of Chemical Biology, Council of Scientific and Industrial Research, Kolkata, India; 2 Institute of Medical Sciences, Benaras Hindu University, Varanasi, India; 3 Institute of Tropical Medicines, Antwerp, Belgium; 4 Calcutta National Medical College and Hospital, Kolkata, India; The Ohio State University, United States of America

## Abstract

**Background:**

In an endeavor to find an orally active and affordable antileishmanial drug, we tested the efficacy of a cationic amphiphilic drug, imipramine, commonly used for the treatment of depression in humans. The only available orally active antileishmanial drug is miltefosine with long half life and teratogenic potential limits patient compliance. Thus there is a genuine need for an orally active antileishmanial drug. Previously it was shown that imipramine, a tricyclic antidepressant alters the protonmotive force in promastigotes, but its in vivo efficacy was not reported.

**Methodology/Principal Findings:**

Here we show that the drug is highly active against antimony sensitive and resistant *Leishmania donovani* in both promastigotes and intracellular amastigotes and in LD infected hamster model. The drug was found to decrease the mitochondrial transmembrane potential of *Leishmania donovani* (LD) promastigotes and purified amastigotes after 8 h of treatment, whereas miltefosine effected only a marginal change even after 24 h. The drug restores defective antigen presenting ability of the parasitized macrophages. The status of the host protective factors TNF α, IFN γ and iNOS activity increased with the concomitant decrease in IL 10 and TGF β level in imipramine treated infected hamsters and evolution of matured sterile hepatic granuloma. The 10-day therapeutic window as a monotherapy, showing about 90% clearance of organ parasites in infected hamsters regardless of their SSG sensitivity.

**Conclusions:**

This study showed that imipramine possibly qualifies for a new use of an old drug and can be used as an effective orally active drug for the treatment of Kala-azar.

## Author Summary

The disease Kala-azar or visceral leishmaniasis is still a big problem in the Indian subcontinent. The antimonials were used for the chemotherapy of Kala-azar but with time its efficacy has reduced dramatically. The newer version of orally active drug miltefosine has been introduced, but its efficacy has decreased considerably as relapse cases are on the rise. Other drugs like liposomal form of amphotericin B is expensive and the patients require hospitalization. Thus there is a genuine need for an orally active antileishmanial drug. There are reports that the cationic amphiphilic molecule, imipramine, a drug used for the treatment of depression in humans, kills the promastigotes of *Leishmania donovani*. We tested the efficacy of imipramine in experimental infection in hamster and mouse model. Our study showed that the drug is highly effective against antimony sensitive and antimony resistant *Leishmania donovani* infected hamsters as well as mouse and offered almost sterile cure.

## Introduction

The disease visceral leishmaniasis or Kala-azar is caused by the protozoan parasite *Leishmania donovani* (LD) and is widening its base in different parts of the world [1], [2]. Pentavalent antimonial or SSG, which has long been the first line drug, is no longer recommended for use as high levels of resistance in the Indian subcontinent have been reported [3]. Other drugs like miltefosine (hexadecylphosphocholine, a polyene antibiotic) and amphotericin B (an anti-fungal agent) are in current clinical use. As miltefosine is orally active, it offers advantages in terms of reduced hospitalization but cannot be used during pregnancy and lactation [2]. Amphotericin B and its liposomal form are to be administered as an infusion and therefore the patients require hospitalization [4]. Unfortunately, treatment failure cases to miltefosine [5] and amphotericin B [6] are emerging, which raises serious concerns for their future use. There is a genuine need for an orally active and affordable drug for the treatment of relapsed Kala-azar cases.

Imipramine, N-(γ-dimethylaminopropyl)-iminodibenzyl HCl, is a tricyclic antidepressant and belongs to the broad class of cationic amphiphilic drugs. The tricycle consists of two benzene rings fused with a seven member heterocycle. Imipramine is FDA (Food and Drug Administration) approved drug for treating depression and paediatric nocturnal enuresis [7], and is sometimes used off-label to treat chronic pain in combination with other pain medications [8]. The dose range for treating depression is 100–200 mg daily and the recommended use for enuresis is 10–75 mg daily [9]. The selection of imipramine for therapy of experimental visceral leishmaniasis is based on the following past observations by others: (i) the drug alters the proton motive force of LD's membrane [10], (ii) inhibits trypanothione reductase, an enzyme upregulated in SSG resistant LD parasites [11], (iii) an effective immunomodulator as it induces the production of TNF-α, an important cytokine for antileishmanial defense [12], (iv) cationic properties favor its absorption by phagocytic cells and accumulation in phagolysosomal bodies [13], and (v) its metabolite desipramine is as effective as the parent drug *against* LD promastigotes [14]. These compelling attributes of imipramine towards *Leishmania* parasites led us to test its efficacy directly on LD and also in experimental infection induced by recent clinical isolates of SSG-S and SSG-R LD parasites with miltefosine as a reference oral drug.

In this investigation, we endeavored to study the effect of oral administration of imipramine in LD infected hamster model. Our study done in hamster model very clearly showed that this drug is highly active in vitro as well as in vivo. Furthermore it plays a strong immunomodulatory role which also favored parasite clearance. Thus imipramine may be used orally in the treatment of visceral leishmaniasis. To our knowledge this is the first report on the therapeutic efficacy of imipramine in experimental visceral leishmaniasis.

## Materials and Methods

### Animals

BALB/c mice (*Mus musculus*) and hamsters (*Mesocricetus auratus*) were maintained and bred under pathogen free conditions. Use of both mouse and hamster was approved by the Institutional Animal Ethics Committees of Indian Institute of Chemical Biology, Kolkata, India. All experiments were performed according to the National Regulatory Guidelines issued by CPSEA (Committee for the Purpose of Supervision of Experiments on Animals), Ministry of Environment and Forest, Government of India.

### Parasite

All parasites for this study were received from European Union KaladrugR project consortium. These parasite samples are fully anonymized and study with these parasites is approved by Institutional Review Board of Institute of Medical Sciences, Benaras Hindu University, Varanasi, India. The details of the patients and the treatment profile of the patients from whom *Leishmania donovani* (LD) parasites were derived have been published previously [15]. Clonal population of LD parasites MHOM/IN/10/BHU816/1 (BHU 816) and MHOM/IN/09/BHU777/0 (BHU 777) are SSG sensitive (SSG-S) and strains MHOM/IN/09/BHU575/0 (BHU 575), MHOM/IN/10/BHU782/0 (BHU 782), MHOM/IN/10/BHU814/1 (BHU 814) and MHOM/IN/10/BHU872/6 (BHU 872) are SSG resistant (SSG-R). LD promastigotes were maintained in M199 medium (Sigma Aldrich, St. Louis, MO) supplemented with 10% heat inactivated FBS (Gibco), 100 IU/mL of penicillin and 100 µg/mL of streptomycin (Gibco) in a 22°C room as described elsewhere [15].

### Preparation of drug stocks for drug assays

Imipramine hydrochloride (Sigma Aldrich, St. Louis, MO) and miltefosine (Kindly provided by Aeterna Zentaris GmbH (Germany) to the KaladrugR project consortium, batch#1149149) solutions were prepared at 1 mg/ml in PBS (Sigma Aldrich, St. Louis, MO), followed by sterile filtration using 0.22 µM filters (Milipore) as and when required.

### Isolation of peritoneal exudate cells (PECs) of BALB/c mice and infection with *Leishmania donovani*

PECs were harvested from BALB/c mice by lavage, 48 h after i.p. injection of 2% (w/v) soluble starch (Sigma Aldrich, St. Louis, MO). For convenience, PECs of BALB/c mice were defined as MΦ. MΦ were harvested on sterile 22 mm square coverslips (Bluestar, India) in 35 mm disposable petriplates (Tarsons, India) at a density of 10^5^/cover slip in RPMI 1640 medium (Sigma Aldrich, St. Louis, MO) supplemented with 10% heat inactivated FBS, 100 IU/mL of penicillin, and 100 µg/mL of streptomycin, i.e. RPMI complete medium. The cells were left to adhere for 48 h at 37°C under 5% CO_2_ before infection. The MΦs were infected with stationary phase promastigotes at a ratio of 1∶10 [14], [15]. After incubating the cultures at 37°C and 5% CO_2_ overnight or for 4 h, non-phagocytosed promastigotes were washed off with serum free medium RPMI 1640 and treatment provided as described [16].

### Toxicity of imipramine on MΦ *in vitro*

MΦs were harvested on a 96-well tissue culture plate (BD Biosciences) in RPMI complete media and left to adhere for 48 h at 37°C under 5% CO_2_. Successive increasing concentrations of imipramine were added in triplicate and incubated for 24 h. After completion of incubation, MTT (Sigma Aldrich, St. Louis, MO) was added and incubated for 4 h at room temperature. Solublizing agents [0.04 N HCl (Merck) in isopropanol (Merck)] were added after incubation and the optical density (OD) was measured after 30 min in a plate reader at 570 nm. The relative number of live cells was determined based on the optical absorbance of the treated and untreated samples and of blank wells, as described previously [17].

### Determination of drug efficacy of imipramine on promastigotes and intracellular amastigotes: IC_50_ and EC_50_

Day 5 culture of parasites was used to determine the drug efficacy (IC_50_) to kill promastigotes using MTT [18]. The LD parasites were plated on the 96-well cell culture plates at a density of 10^5^ cells/well and kept in presence of imipramine for 48 h. Results were expressed as the concentration that inhibited parasite growth by 50% (IC_50_). Analysis was carried out using Graphpad Prism5 software (version 5.03).

In order to determine EC_50_ (Efficacy against intracellular amastigote), the drug was serially diluted in RPMI complete medium over six concentrations in triplicate at each concentration. Stock solutions and dilutions were freshly prepared for each use. Infected MΦs were incubated with drug dilutions for another 24 h at 37°C and under 5% CO_2_. Untreated MΦs received medium alone and intracellular parasites were enumerated.

At the endpoints, the coverslips were washed with PBS, dried, fixed with 100% methanol (Merck), stained with 10% Giemsa (Sigma Aldrich, St. Louis, MO) and examined microscopically. One hundred MΦs/coverslip were scored and the amastigotes were enumerated [19]. The average of three untreated cultures was taken as 100% control against which the percentage inhibition of infected MΦs in treated cultures was calculated. The 50% effective concentration (EC_50_) of imipramine for each of the isolates was estimated as described elsewhere [19], [20].

### Mitochondrial transmembrane potential determination

The JC-1 dye (Molecular Probes, Eugene, OR) has been used routinely to monitor the mitochondrial potential [21]. The monomeric form has an emission maximum at 527 nm. The dye at higher concentrations or potentials forms red fluorescent J-aggregates with an emission maximum at 590 nm. The ratio of this red/green (λ_590_/λ_527_) fluorescence is known to depend only on the membrane potential. A working solution of JC-1 was therefore prepared as per manufacturer's instruction. Imipramine and miltefosine treated and untreated LD promastigotes were incubated with the JC-1 working solution for 25 min in a 96-well plate and washed. Cell pellets were resuspended in assay buffer and analyzed under a fluorescent plate reader (Fluorescence plate reader LS 55, Perkin Elmer).

### Detection of phosphatidylserine exposure

Double staining for annexin V fluorescein isothiocyanate (FITC)-PI was performed with the Annexin-V apoptosis detection kit (Molecular Probes, Eugene, OR) [22]. In brief, untreated, imipramine-treated, or miltefosine-treated promastigotes were washed twice in cold PBS and centrifuged at 3000 rpm for 10 min. The pellets were resuspended in 100 µL of annexin V-FITC in the presence of PI according to the instructions of the manufacturer. After 15 min of incubation in the dark, the intensity of annexin V-FITC labeling was recorded on a flow cytometer (FACSARIA II, Becton Dickinson, San Diego, CA) and analyzed with FACSDIVA software, version 6.1.1; the percentage of positive cells was then assessed.

### Fluorescence anisotropy (FA) determination

The membrane fluorescence and lipid fluidity of MΦ under parasitized condition as well as after imipramine treatment were measured following the method described by Shinitzky and Inbar [23]. Briefly, the fluorescent probe DPH (Molecular Probes, Eugene, OR) was dissolved in tetrahydrofuran (Merck) at 2 mM concentration. To 10 ml of the rapidly stirring PBS solution (pH 7.2), 10 µL of 2 mM DPH solution was added. For labeling, 10^6^cells were mixed with an equal volume of DPH in PBS (c*f* 1 µM) and incubated for 2 h at 37°C. Thereafter the cells were washed thrice and resuspended in PBS. The DPH probe bound to the membrane of the cell was excited at 365 nm and the intensity of emission was recorded at 430 nm in a spectrofluorometer (LS 55, Perkin Elmer). The FA value was calculated using the equation: FA = [(I_II_−I_⊥_)/(I_II_+2I_⊥_)], where I_II_ and I_⊥_ are the fluorescent intensities oriented, respectively, parallel and perpendicular to the direction of polarization of the excited light [23].

### Antigen presentation assay

MΦs, also defined as antigen presenting cells (APCs), were harvested from peritoneal cavity of mice at 10^6^ cells/well in a 48 well tissue culture plate, then incubated for 24 h with specific peptide Lambda repressor λR_12–26_ (GenScript, USA) and T cell hybridoma 9H3.5 (kind gift of Professor Malcolm Gefter, Massachusetts Institute of Technology, Cambridge, Massachusetts) in complete RPMI medium in a 37°C incubator. The culture supernatants were analyzed for the presence of IL 2 using mouse IL 2 ELISA kit (BD Biosciences, San Diego, CA) as per manufacturer's instruction.

### Measurement of super oxide and nitric oxide

To monitor the level of reactive oxygen species (ROS, including superoxide, hydrogen peroxide, and other reactive oxygen intermediates), the cell-permeable, non polar, H_2_O_2_-sensitive probe H_2_DCFDA (Molecular Probes, Eugene, OR) was used [24]. The extent of H_2_O_2_ generation was defined as the extent of ROS generation for convenience. For each experimental sample, fluorometric measurements were performed in triplicate and the results were expressed as the mean fluorescence intensity per 10^6^ cells. Nitric oxide (NO) generation was monitored by using the Griess reagent (Molecular Probes, Eugene, OR) as described previously [25], and the results are expressed in µM nitrite.

### Infection and purification of amastigote

To infect hamsters (6 weeks old), two SSG-S (BHU 777 and BHU 816) and two SSG-R (BHU 575 and BHU 814) LD amastigotes were purified as described [26] and inoculated (10^7^ parasites in 200 µL) via intracardiac routes as described previously [27].

### Selection of imipramine dose for *in vivo* use

Imipramine is usually used in human at a dose of 100–200 mg/day (average of 150 mg/day) for the treatment of depression [9], [28]. Considering the average human body weight of 60 kg, the effective dose is 2.5 mg/kg/day. Based on the dose equivalence between human and rodents [29], the dose of imipramine in mouse and hamsters would be 41 and 25 mg/kg/day respectively. In our investigation, the highest dose used was 5 mg/kg/day both in mouse and hamsters which is effectively 12.3 and 7.4 times lower than the equivalent human dose. Miltefosine is used in human at the dose 2.5 mg/kg [5]. Based on dose equivalent formula, we converted the normal human dose to hamster equivalent dose. Thus we treated hamsters with the maximum dose 17.5 mg/kg which is ∼7 times high then the normal human dose.

### Oral administration of imipramine and miltefosine in infected hamsters and determination of parasite burden

The 8-week infected hamsters (i.e. 14-week old hamsters) were randomly divided into four groups (groups I to IV). Group I received only saline, groups II to IV received imipramine at the dose levels of 0.05, 0.5 and 5 mg/kg/day respectively for 4 weeks by oral route using a feeding needle as described by others [30]. Miltefosine treatment was carried out in 8-week infected hamsters for 4 weeks at a dose of 17.5 mg/kg/day orally. Two days after the completion of treatment, hamsters were sacrificed to determine splenic and hepatic parasite burdens by stamp smear method as described elsewhere [27], [31], as well as by the serial dilution method [27].

### Collection of blood and preparation of serum

Blood was collected from hamsters and mice as described previously [27] and kept overnight at 4°C; serum was prepared by centrifugation.

### Preparation of soluble leishmanial antigen (SLA)

Soluble Leishmanial Antigen (SLA) was prepared from stationary phase LD promastigotes of LD following the published protocol [27]. Briefly, leishmanial lysate from washed promastigotes (10^9^/ml) was prepared by several cycles (minimum six) of freezing (−70°C) and thawing (37°C) followed by 5 min incubation on ice. Partially lysed promastigotes were then disrupted in a sonicator (Misonex, Farmingdale, NY) thrice for 30 s each and centrifuged at 10,000 rpm for 30 min at 4°C. The supernatant containing soluble antigen was collected and the protein concentration was determined by Bradford Protein Assay method (Bio-Rad, Herculis, CA). The prepared antigen was stored at −70°C until further use.

### T-cell proliferation assay

Splenocytes from different experimental groups of hamsters were prepared after Ficoll (Sigma Aldrich, St. Louis, MO) density gradient centrifugation and then suspended in complete RPMI medium. Cells were plated in triplicate at a concentration of 10^5^ cells/well in 96-well plates and allowed to proliferate for 3 days at 37°C in a 5% CO_2_ incubator either in the presence or absence of SLA (5 µg/ml) (29). For ConA (Sigma Aldrich, St. Louis, MO) induced proliferation, the mitogen was added at a concentration of 5 µg/mL as described previously [27]. Cells were treated with MTT (0.5 mg/mL) 4 hr before harvest as described previously [18] and incubated again at the same condition for 4 more hrs. MTT crystals were then solubilised using Isopropanol-HCl mixture (0.04%) and the absorbance at 570 nm was read at an ELISA plate reader (DTX 800 multimode detector, Beckman Coulter, California).

### Measurement of antileishmanial antibody response in hamsters

Serum samples were obtained from different groups of hamsters and mice (five animals per group), and analyzed to determine the parasite SLA-specific antibody titer. 96-well Enzyme-Linked ImmunoSorbent Assay (ELISA) plates were coated with SLA (2 µg/ml) in PBS for overnight at 4°C. The plates were blocked with 5% FCS in PBS at room temperature for 1 h to prevent nonspecific binding. Sera from different groups of hamsters were added at various dilutions, and incubated for 2 h at room temperature. These were diluted 10^−1^, 10^−2^, and 10^−3^ times for the determination of IgG1 and 10^−3^, 10^−4^, and 10^−5^ times for IgG2. Biotin-conjugated mouse anti-hamster IgG1 (BD Biosciences, San Diego, CA) and mouse anti-Armenian and anti-Syrian hamster IgG2 (BD Biosciences, San Diego, CA) were added and incubated for 1 h at room temperature; this was followed by 1 h of incubation with the detection reagent (streptavidin-conjugated horseradish peroxidase). As a peroxide substrate in citrate buffer (0.1 M, pH 4), TMB (Sigma) was added along with 0.1% H_2_O_2_ (Merck) to a 96-well plate, and the absorbance at 450 nm was read with an ELISA plate reader [27].

### RNA isolation and semiquantitative RT-PCR analysis of cytokines and iNOS

RNA was isolated from the splenocyte of hamsters using Trizol (Invitrogen) as described previously [27]. The forward and reverse primers were used to amplify cytokine transcripts. All of these hamster-specific primers, except for the inducible NO synthase (iNOS) primer, were originally described by Melby et al. [31]. The following forward and reverse primers were used: for IL 10, forward primer 5′ACAATAACTGCACCCACTTC3′ and reverse primer 5′AGGCTTCTATGCAGTTGATG3′ (432-bp product); for IL 4, forward primer 5′CATTGCATYGTTAGCRTCTC3′ and reverse primer 5′TTCCAGGAAGTCTTTCAGTG3′ (463-bp product); for interferon gamma (IFN γ), forward primer 5′GGATATCTGGAGGAACTGGC3′ and reverse primer 5′CGACTCCTTTTCCGCTTCCT3′ (309-bp product); for tumor necrosis factor alpha (TNF α), forward primer 5′GACCACAGAAAGCATGATCC3′ and reverse primer 5′TGACTCCAAAGTAGACCTGC3′ (695-bp product) and for transforming growth factor beta (TGF-β), forward primer 5′CCCTGGAYACCAACTATTGC3′ and reverse primer 5′ATGTTGGACARCTGCTCCAC3′ (310-bp product). To obtain specific amplification for iNOS, the following specific primers were used (6): forward primer 5′ GCAGAATGTGACCATCATGG3′ and reverse primer 5′CTCGAYCTGGTAGTAGTAGAA3′ (198-bp product). For hypoxanthine phosphoribosyl transferase (HPRT) amplification the following primers were used [31]: forward primer 5′ATCACATTATGGCCCT CTGTG3′ and reverse primer 5′CTGATAAAATCTACAGTYATGG3′ (125-bp product). Degenerate bases are indicated above by International Union of Pure and Applied Chemistry designations (Y = C or T; R = A or G).

Details of the procedure have been described previously [27]. Densitometry analyses were done using the ImageJ software (v1.41o), ethidium bromide staining, and visualization under a UV transilluminator. For densitometric calculations, the same band area was used to determine band intensity and normalized for HPRT.

### Survival kinetics

To evaluate long-term therapeutic ability, normal hamsters, infected hamsters and imipramine treated infected hamsters (30 hamsters per group) were used to study survival kinetics as described previously [32].

### Histological studies

Spleens and livers were fixed in 10% formalin (Merck) and embedded in paraffin. Tissue sections (5 µm) were stained with hematoxylin-eosin to study their microarchitecture by light microscopy. Photomicrographs were taken with a Nikon Eclipse E200 microscope.

### Statistical analysis

The statistical significance of differences between groups was determined by the unpaired two-tailed Student's *t* test. Statistical significance was defined as a *P* value of <0.05 and the results were expressed as averages and standard deviations of triplicate measurements.

## Results

### IC_50_ and EC_50_ of recent clinical isolates towards imipramine

Details on the clinical isolates used in this investigation in terms of their sensitivity to Sodium stibogluconate (SSG) have already been published [15]. Out of these, two SSG sensitive (BHU 777 and BHU 816) and four SSG resistant strains (BHU 575, BHU 782, BHU 814 and BHU 872) were selected for this investigation. These isolates were subjected to imipramine treatment *in vitro* and *ex vivo* to measure the IC_50_ and EC_50_ (Table 1). It was observed that regardless of difference in SSG sensitivity, there was no significant difference in IC_50_ or EC_50_ (Table 1). For convenience, the rest of the study was carried out with two SSG-S (BHU 777 and BHU 816) and two SSG-R (BHU 814 & BHU 575) isolates, and were defined as BHU 816(S), BHU 777(S), BHU 814(R) and BHU 575(R) respectively.

**Table 1:** IC_50_ and EC_50_ values of the clinical LD isolates.

Isolate Code	Imipramine (µM)	
	IC_50_	EC_50_
**BHU 872 (R)**	3.89±0.06	22.23±0.59
**BHU 782 (R)**	3.77±0.17	21.01±1.13
**BHU 814 (R)**	4.00±0.31	21.02±0.19
**BHU 575 (R)**	3.83±0.24	22.63±2.10
**BHU 777 (S)**	3.72±0.12	22.38±0.98
**BHU 816 (S)**	3.68±0.09	22.42±0.98

IC_50_ and EC_50_ values of imipramine to recent antimony sensitive (BHU 777 and BHU 816) and antimony resistant (BHU 872, BHU 782, BHU 814 and BHU 575) clinical isolates of *Leishmania donovani* (LD) promastigotes.

### Imipramine decreases the mitochondrial transmembrane potential and induces early apoptosis of LD parasites

**Figure 1:**
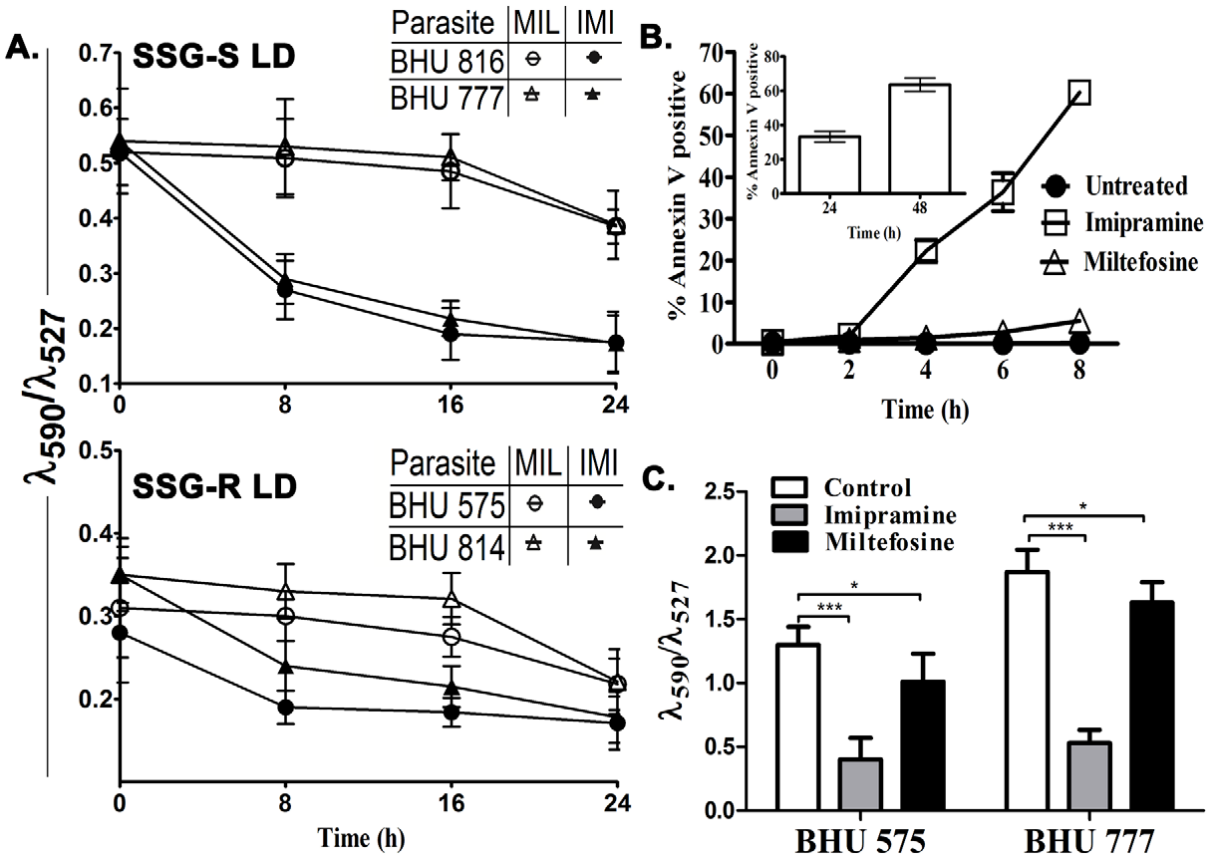
Imipramine and miltefosine induced alteration of mitochondrial transmembrane potential and apoptosis induction in LD promastigotes. A. BHU 816(S), BHU 777(S), BHU 575(R) and BHU 814(R) promastigotes were exposed to 75 µM imipramine (•,▴) or 40 µM miltefosine (○,Δ) for 8, 16 and 24 h and the mitochondrial transmembrane potential was determined using JC-1 fluorescent probe. The fluorescence was measured at 527 and 590 nm and the ratio (**λ_590_/λ_527_**) was plotted. Three independent experiments were performed and mean±SE was presented. B. Apoptosis of LD parasites as a measure of % Annexin V positive cells in BHU 575(R) in the presence of 75 µM imipramine (□) or 40 µM miltefosine (Δ) and absence of drug (•) was measured as a function of time. Phosphatidylserine exposure was analyzed by flow cytometry. Inset 1B showing the percentage of apoptotic cells in BHU 575(R) upon miltefosine (40 µM) treatment at 24 and 48 hour. The data are the mean of three independent experiments, and standard deviations are represented by error bars. C. BHU 777(S) and BHU 575(R) amastigotes were subjected to either 75 µM imipramine or 40 µM miltefosine treatments for 8 h and the resulting mitochondrial transmembrane potential was measured using JC-1 as above.

The transmembrane potential (ΔΨ_m_) was evaluated using JC-1, a lipophilic cationic dye as described [21]. For this investigation the drugs imipramine and miltefosine were used at a concentration of 75 µM [33] and 40 µM [22] respectively. We observed that both the SSG-R and SSG-S strains showed similar sensitivities to imipramine as evident from the significant decrease in ΔΨ_m_ after 8 h of treatment (Figure 1A). On the other hand, miltefosine failed to induce any change in ΔΨ_m_ at 8 h of treatment (Figure 1A). After 8 h of imipramine exposure to BHU 575(R), 60% of parasites were apoptotic whereas miltefosine induces apoptosis in only 5.5% parasites (Figure 1B). However at 24 h and 48 h after miltefosine treatment the extent of apoptotic BHU 575(R) was 32.8% and 60.7% respectively (Inset Figure 1B). Similar studies were performed with lesion derived purified amastigotes, BHU 575(R) and BHU 777(S) to find that 8 h treatment with imipramine, but not miltefosine induced a significant decrease in ΔΨ_m_ (Figure 1C).

### Imipramine clears intracellular parasites *ex vivo*

**Figure 2:**
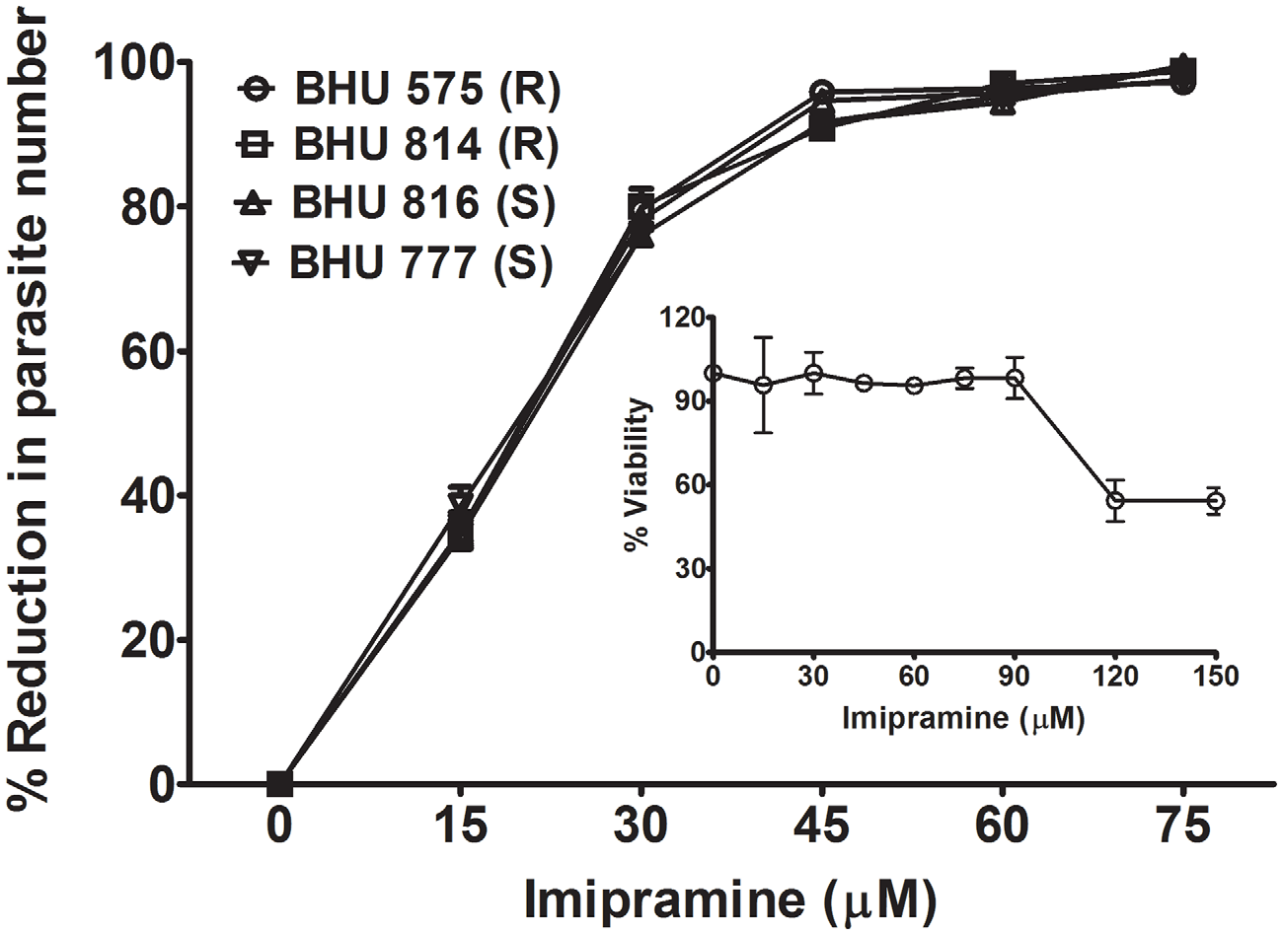
Imipramine clears intracellular parasites in *in vitro* infected macrophage culture. BALB/c derived MΦ were infected with either BHU 575(R), or BHU 814(R), or BHU 816(S) or BHU 777(S) for 24 h and treated with imipramine at increasing doses for 48 h. Intracellular parasite numbers/100 MΦ were calculated after Giemsa staining and data represented as % reduction with respect to the infected control. Inset showing viability of normal MΦ measured as a function of imipramine concentration for 48 h. The viability of MΦ was determined using the MTT assay. Three experiments were performed 3 times and mean±standard error was plotted.

The replication of intracellular LD in the presence of imipramine was studied in *in vitro* infected MΦ. It was observed that intracellular LD replication was inhibited very efficiently as a function of the imipramine concentration regardless of the SSG sensitivity (Figure 2). The dose required to clear 100% of the intracellular parasites was around 60 µM of imipramine. To show that 60 µM of the drug has no toxic effect; MΦs were incubated with increasing concentration of imipramine. It was observed that almost 100% MΦs remained viable upto 90 µM imipramine (Figure 2, inset).

### Imipramine treatment restores the membrane rigidity and antigen presenting ability of infected MΦ

**Figure 3:**
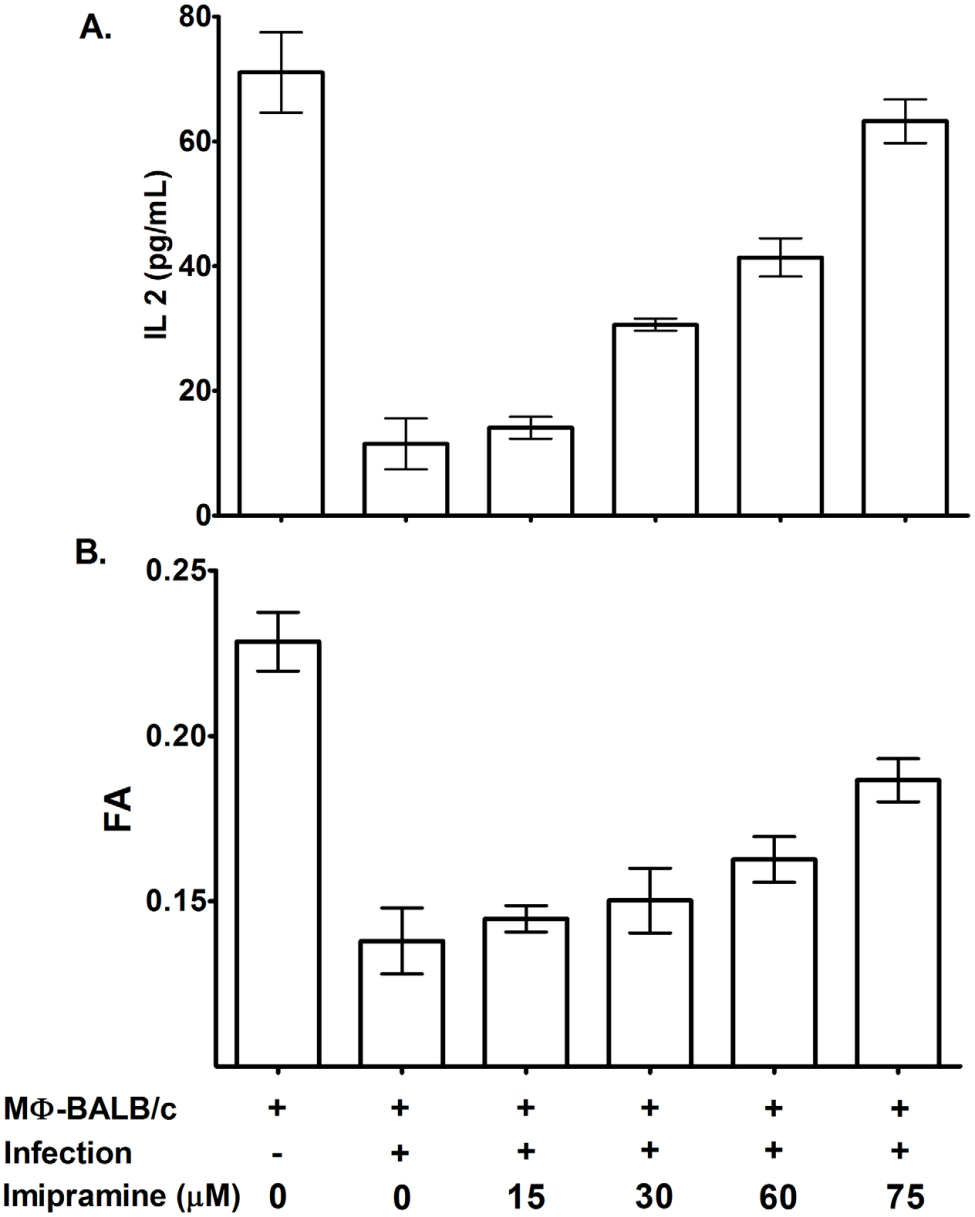
Imipramine treatment of PECs increased membrane fluidity and enhanced T cell stimulating ability. A. Infected PECs of BALB/c origin were treated with increasing dose of imipramine and the resulting fluorescent anisotropy (FA) was measured using DPH as a probe. B. The T cell stimulating abilities of normal, infected and imipramine treated infected PECs were studied using A^d^ restricted T cell hybridomas 9H3.5 in the presence of appropriate peptide (Lambda repressor) and the resulting IL 2 production was assayed by ELISA. These are the result from 3 independent experiments and Mean±SE was shown.

It is known that infected MΦs are more fluid than their normal counterpart, and this is associated with defective T cell stimulating ability [32]. For convenience BHU 575 (R) infected MΦ were defined as MΦ-575 (R). To show that imipramine restores membrane rigidity, we treated MΦ-575 (R) with increasing dose of imipramine and observed that there was a gradual increase in fluorescence anisotropy (FA) value in a dose dependent manner (Figure 3A). To show that imipramine treatment restores the antigen presenting ability, MΦ-575 (R) were used as antigen presenting cells (APC) with and without imipramine treatment. This showed that the T-cell stimulating ability of MΦ-575 (R) is improved as a function of imipramine concentration as evident from the increase in resulting IL-2 production from I-A^d^ restricted T-cell hybridoma (Figure 3B).

### Imipramine activates normal MΦs to generate superoxide and nitric oxide

**Figure 4:**
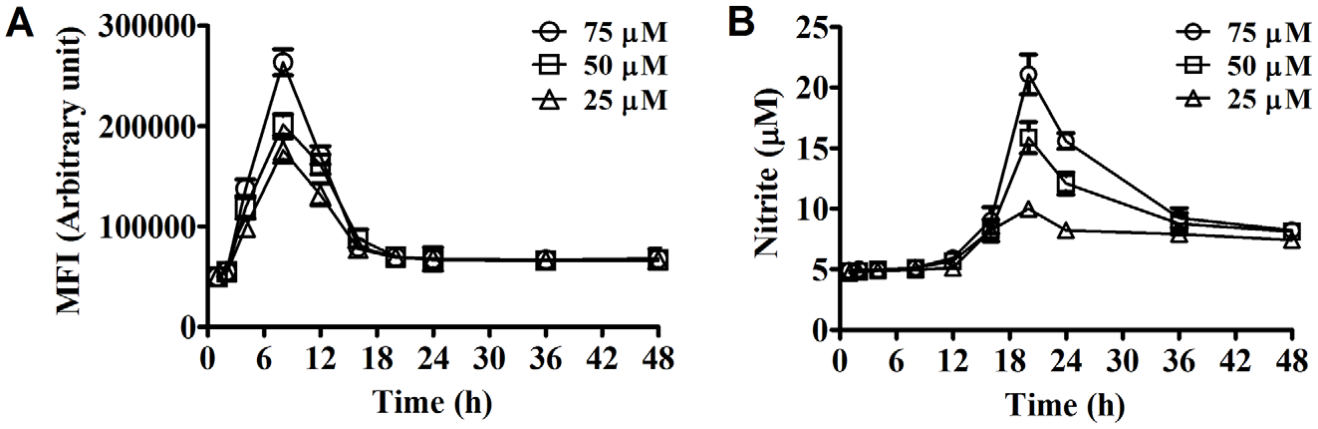
Generation of ROS and NO from MΦs. Time kinetics as well as concentration dependence of ROS (A) and NO (B) generation from normal BALB/c derived peritoneal MΦ upon imipramine treatment was studied. Three independent experiments were performed and standard error mean was plotted.

ROS (Reactive Oxygen Species) and NO (Nitric oxide) are two very important leishmanicidal molecules [34]. Generation of these molecules was found to be enhanced in a time and dose dependent manner in imipramine treated MΦs (Figure 4). ROS generation reached a plateau at around 8 h in the presence of 75 µM imipramine treatment (Figure 4A), whereas maximum NO generation was observed after 20 h exposure at the same concentration of imipramine (Figure 4B).

### Oral administration of imipramine but not miltefosine completely clears organ parasites in infected mice and hamsters

**Figure 5:**
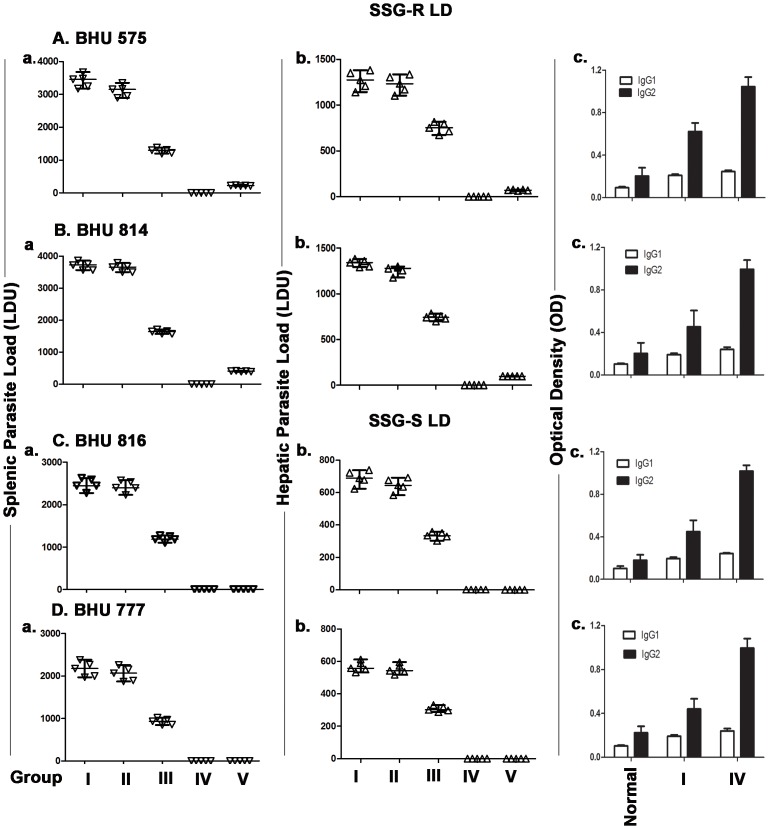
Oral administration of imipramine clears hepatic and splenic parasites in infected hamsters. Six weeks old hamsters were infected with either BHU 575(R), or BHU 814(R) (Fig 5A and 5B), or BHU 816(S), or BHU 777(S) (Fig 5C & 5D) LD parasites and infection was allowed to establish for next 8 weeks. Eight-week infected hamsters received the following doses of imipramine 0.05 mg/kg/day (Group II), 0.5 mg/kg/day (Group III) and 5 mg/kg/day (Group IV) respectively orally on a daily basis for 4 weeks. Group I hamsters were infected group received saline only. Similarly eight-week infected hamsters received miltefosine at the dose of 17.5 mg/kg/day for 4 weeks (Group V). Two days after the last treatment hamsters were sacrificed and the hepatic as well as the splenic parasite load was determined by stamps-smear method and the by limiting dilution method. Total parasite load in each organ is expressed in LDU unit. 1 LDU = amastigote per nucleated cell×organ weight in milligram. Fig 5(A–D) c shows the antileishmanial IgG1 and IgG2 levels in normal, untreated infected (Group II) and imipramine treated infected hamsters (Group IV). Antibody titer was measured by ELISA. Experiments were repeated thrice and one representative data was shown. The error within the experiments was within 10%.

The effect of graded doses of orally administered imipramine on the splenic and hepatic parasite load in infected hamsters was investigated. Hamsters were infected with BHU 816(S), BHU 777(S), BHU 814(R), or BHU 575(R) LD isolates. We performed a microscopic evaluation of stamp smears and limiting dilutions to detect parasites in tissue samples of infected organs. Eight-week infected hamsters were divided into 4 groups (I–IV) for a given isolate. Groups I–IV received imipramine at doses of 0, 0.05, 0.5 and 5 mg/kg/day respectively for 4 weeks. The results were expressed as total parasite load in terms of LDU. There was no clearance of splenic and hepatic parasite load in group ΙΙ whereas about 50% clearance was observed in group ΙΙΙ animals and there were no detectable parasites in group ΙV animals (Figure 5 A–D). The organ parasite clearance essentially showed similar trends after imipramine treatment for all the isolates regardless of their SSG sensitivity.

To show that group IV hamsters were indeed infected with LD, antileishmanial antibodies were measured in the animals. The presence of antileishmanial IgG2 antibodies in the sera of these animals was detected together with a marginal increase in anti IgG1 titer (Figure 5 A–D c).

We used miltefosine as a reference drug and studied its effect on the organ parasite clearance in a similar set up. Here also eight-week infected hamsters were subjected to miltefosine treatment (17.5 mg/kg/day) orally for 4 weeks and splenic and hepatic parasite load were determined 2 days after completion of the last treatment dose (Figure 5 A–D Group V). The dose of miltefosine was selected as described elsewhere [5]. This showed that miltefosine treated hamsters infected with either BHU 575(R) or BHU 814(R) had low level of residual parasites in the spleen and liver (Figure 5 A–B) whereas miltefosine treated hamsters infected with BHU 816(S) or BHU 777(S) showed no residual parasites (Figure 5 C–D). The presence of residual parasites was further confirmed by limiting dilution experiments with spleen tissue (Figure S1).

### Imipramine treatment favors the expression of antileishmanial T cell repertoire of infected hamsters

**Figure 6:**
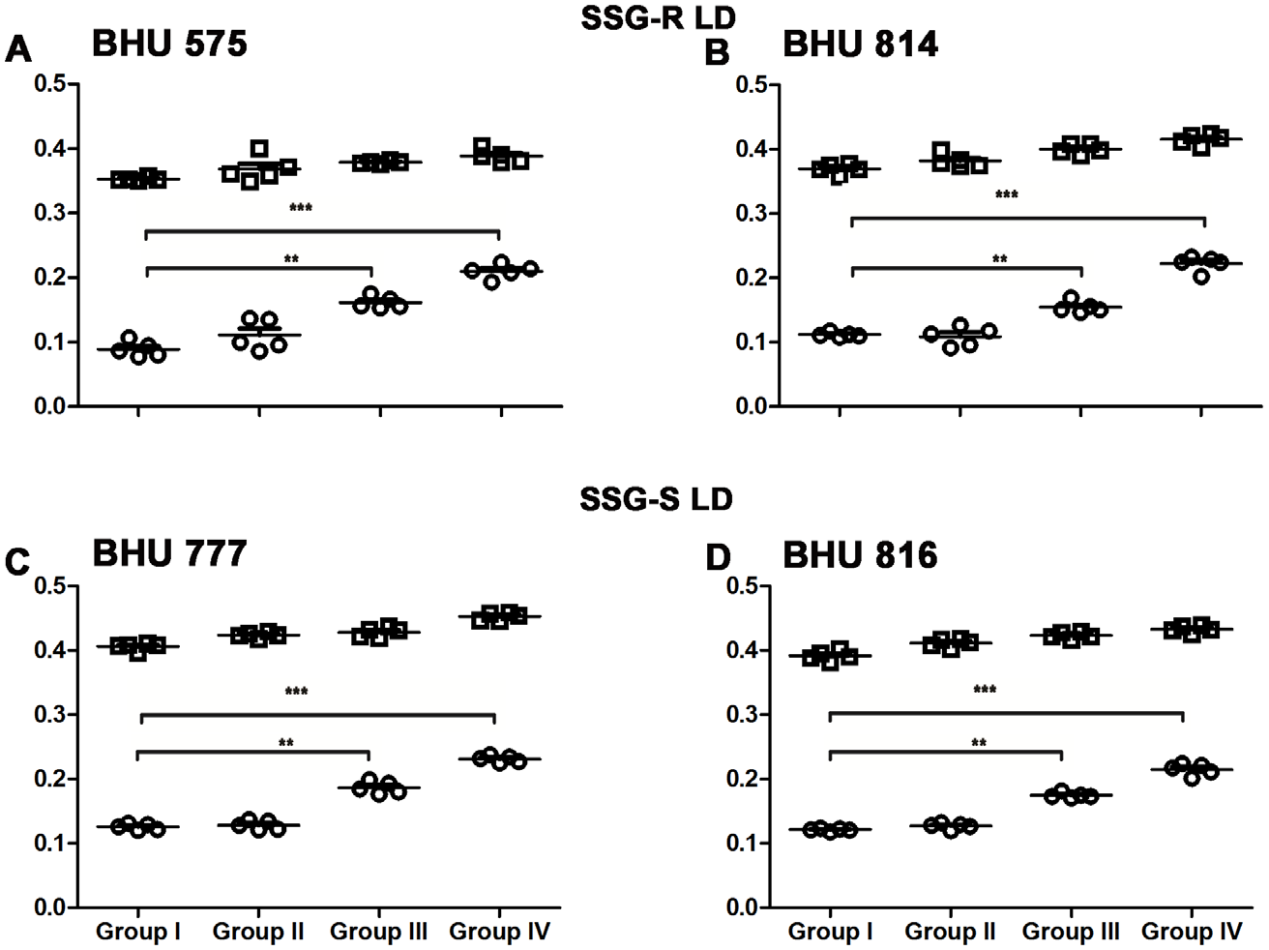
Imipramine treatment favors expansion of antileishmanial T cells. Splenocytes isolated from SSG-R (Figure A, B) and SSG-S (Figure C, D) LD infected (Group I), and 0.05 mg/kg/day (Group II), 0.5 mg/kg/day (Group III) and 5 mg/kg/day (Group IV) drug treated animals were stimulated with SLA (5 µg/mL) (○) as well as non specific mitogen ConA (5 µg/mL) (□), and the resulting proliferation of splenocytes was assayed using MTT cell viability assay. Experiments were repeated thrice one representative data was shown.

To study the status of antileishmanial T cell repertoire in infected and imipramine treated infected hamsters, splenocytes were purified and stimulated either with SLA or ConA. The hamsters were infected either with SSG-S (BHU 816 and BHU 777) or SSG-R (BHU 814 and BHU 575) LD parasites. After completion of imipramine treatment in 8 week infected hamsters, animals were sacrificed and splenocytes were prepared. Fixed concentrations of SLA and ConA were used to stimulate splenocytes as described previously [27]. Splenocytes of group ΙΙ (received 0.05 mg/kg body weight) hamsters failed to mount any antileishmanial immune response but responded well to non specific mitogen ConA regardless of the phenotype of the input parasites for infection (Figure 6). The SLA specific proliferation was marginally improved in group ΙΙΙ (received 0.5 mg/kg body weight/animal), but was further improved in group ΙV (receiving 5 mg/kg body weight/animal). The antileishmanial T cell response was essentially similar regardless of the phenotype of SSG sensitivity. The response to the non specific mitogen ConA remained unaltered in infected and in imipramine treated animals regardless of the dose of imipramine (Figure 6).

### Imipramine treatment increased IFN-γ, iNOS, and TNF-α levels but decreased IL-10 and TGF-β levels in hamster

**Figure 7:**
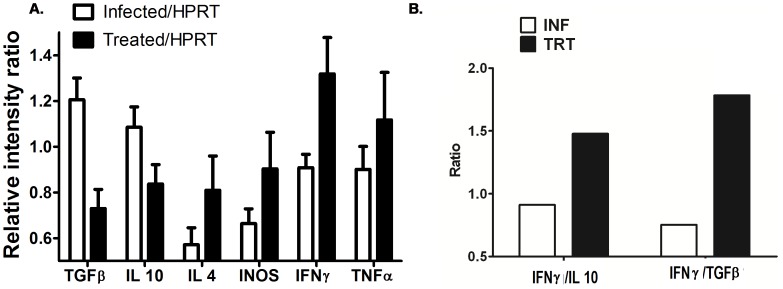
Semiquantitative RT-PCR cytokine, iNOS, and HPRT profiles of LD infected and imipramine treated infected hamsters. Equivalent amounts of RNA from splenic tissues of two groups of hamsters (n = 5) were used as the input for RT-PCR analysis, where the HPRT gene was used as the housekeeping control gene. Expression of each cytokine transcript was expressed as a ratio of cytokine mRNA to HPRT mRNA (Figure A). The values obtained from densitometric analysis of each cytokine were expressed as mean±SE instead of individual values. The ratios of IFN-γ to IL-10 and to TGF-β were determined for infected hamsters and imipramine treated infected hamsters (Figure B).

The ability of cytokine and iNOS gene expression in infected hamsters and imipramine treated infected hamsters was studied by profiling cytokine gene expression (Figure 7A). The results generated from a densitometry analysis of each hamster were collectively expressed as mean±sd for each cytokine, and the statistical significance between groups was determined (Figure 7A). Comparative cytokine analysis showed that the expression of IFN-γ,TNF-α and iNOS transcripts were 1.43, 1.23, and 1.35 times higher, respectively, in imipramine treated hamsters than in infected hamsters, whereas the levels of TGF-β and IL-10 transcripts were 1.65 and 1.13-fold lower, respectively. Studies of the ratio of IFN-γ to TGF-β or IL-10 revealed that the IFN-γ/TGF-β ratio was 1.78-fold greater in imipramine treated hamsters than in infected hamsters (Figure 7B). Similarly, the IFN-γ/IL-10 ratio was 1.47-fold greater in imipramine treated animals than in infected ones (Figure 7B).

### Oral imipramine treatment cured infected hamsters in the long run

**Figure 8:**
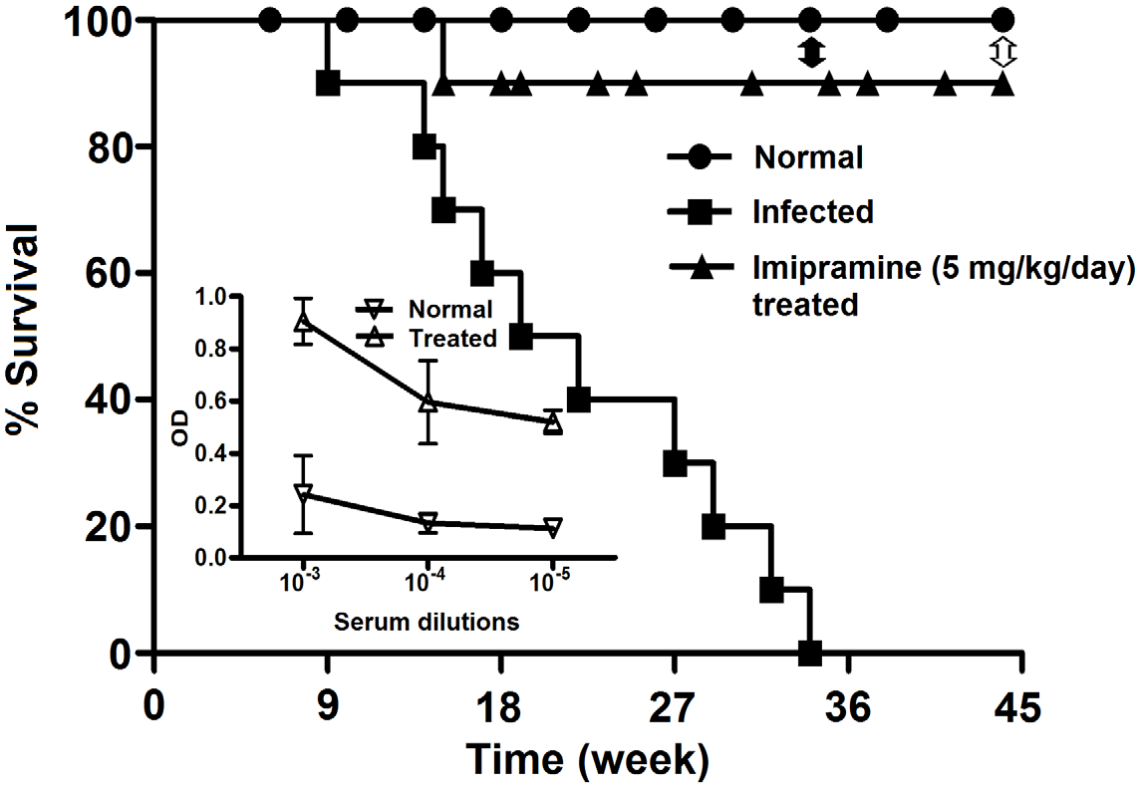
Study of survival kinetics. Long term survival of uninfected (•), BHU 575(R) infected (▪) and imipramine treated infected hamsters (▴) in terms of % survival was studied. Thirty hamsters from each group were used for the investigation. Closed double headed arrow indicates the point when 50% of imipramine treated hamsters were sacrificed (34th) to determine parasite load and antibody titer. Remaining hamsters were sacrificed on 44^th^ week represented by open double headed arrow to determine organ parasites, if any. Inset showing the antileishmanial IgG2 levels of the imipramine treated infected group and normal group at 34^th^ week. The error bars indicate standard deviations. OD is optical density.

To show that imipramine treated hamsters are also protected in the long run, we studied the survival kinetics of infected hamsters and imipramine treated infected hamsters (5 mg/kg/day for 4 weeks), using normal hamsters as control. In each group 30 hamsters were used. Hamsters were infected at 6 weeks and infection was allowed to proceed for another 8 week, i.e. before initiating any treatment. We observed that 80% of the infected hamsters survived up to 14 weeks, 60% up to 18 weeks, 20% up to28 weeks, and the rest died by 34 weeks. On the other hand, 90% of imipramine treated infected hamsters remained alive until the termination of the experiment, i.e. 44 weeks (Figure 8). Remarkably, amastigotes could not be detected by microscopy in impressions of Giemsa-stained tissue stamp smears of spleen and liver or by limiting dilution experiments at 34^th^ week and also at 44^th^ week. The organ weights had returned to near normal (Table 2 lower panel), and high titers of antileishmanial IgG2 persisted at 34^th^ week (Figure 8 inset).

**Table 2:** Organ weight of normal, infected and imipramine treated infected hamsters.

Groups	Organ Weight (gm) at age (weeks)					
	18		34		44	
	Spleen	Liver	Spleen	Liver	Spleen	Liver
**infection**	1.779±0.102	5.280±0.179	1.962±0.126	6.008±0.335	NA	NA
**8 week infection +4 week treatment**	1.205±0.166	4.679±0.335	1.036±0.107	4.031±0.243	0.488±0.984	3.711±0.934
**Age matched normal**	0.327±0.103	1.775±0.123	0.393±0.054	2.964±0.048	0.467±0.523	3.568±0.485

Hepatic and splenic organ weight in gm in untreated uninfected, untreated infected, and imipramine treated infected hamsters. NA = Not available.

### Imipramine treatment induced hepatic granuloma formation in hamster

**Figure 9:**
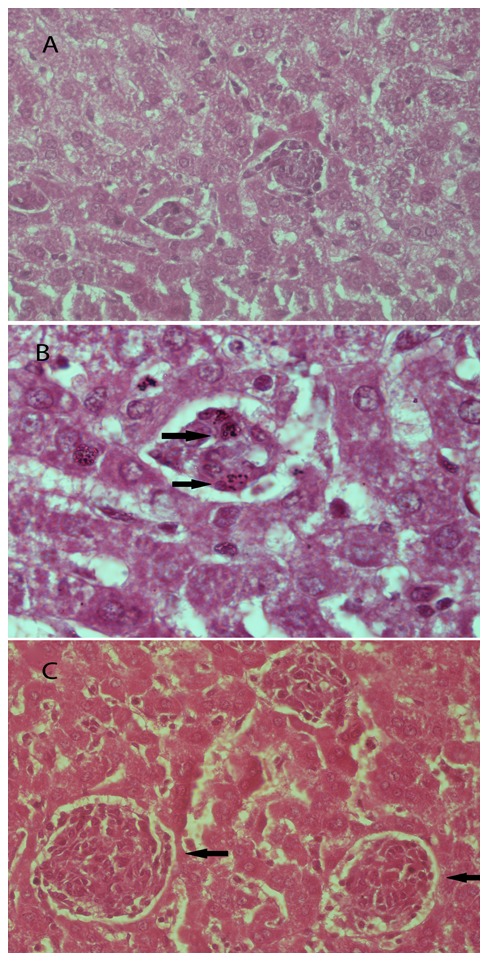
Hepatic histologic response to infected as well as imipramine treated infected hamsters. **A.** Immature hepatic granuloma formation in 12 week post infection (right arrow). B. The same granuloma as in Fig A at 100× magnification showing Kupffer cells harbouring *Leishmania donovani* (right arrow). C. Abundant mature granulomas formation after imipramine treatment (40×) (left arrow).

Evolution of granuloma formation in infected and imipramine treated infected hamsters was studied. Liver section of LD infected hamsters showed immature granuloma formation associated with Kupffer cells surrounded by less number of infiltrating lymphocytes (Figure 9A). High resolution figure of the same showed the presence of parasitized Kupffer cells (Figure 9B) inside the cell assembly. Imipramine treated infected liver tissue shows fair number of lymphocyte infiltration in periportal area and the presence of fair number of mature and uniform granuloma (Figure 9C). Parasites could not be seen in these mature granulomas.

### Organ weight in normal, infected and imipramine treated infected hamsters

The organ weight at 18 weeks increased significantly in untreated infected animals as compared to uninfected animals, but the 4 week imipramine treatment in infected group showed only a marginal decrease in organ weight (Table 2). After 34 weeks, the organ weight continued to increase marginally in the untreated infected group whereas in the imipramine treated group the organ weight was reduced by 50% as compared to the 18 week time point. However, at 44 weeks the organ weight of the imipramine treated infected group was similar to that of the age matched normal (Table 2).

## Discussion

In an effort to find new orally active chemotherapeutics for visceral leishmaniasis, we evaluated the potential of the existing antidepressant drug imipramine against SSG-R and SSG-S LD parasites. The potent in vitro activity of imipramine against intracellular amastigotes (EC_50_ = 16.2 µM) [14] as well as promastigotes (Low IC_50_ values, Table 1) coupled with the absence of obvious cytotoxicity on host MΦ formed the basis for advanced exploration of this promising lead.

Our study showed that imipramine decreases the mitochondrial transmembrane potential of SSG-S and SSG-R LD promastigotes significantly by 8 h of treatment which continued to decrease with time. In contrast, miltefosine showed only a marginal change in the mitochondrial transmembrane potential only at 24 h of treatment. Similar observation was made in imipramine or miltefosine treated purified amastigotes. Oddly enough, imipramine induced 60% of apoptosis of LD promastigote at 8 h whereas miltefosine at that stage induced only 5.5% apoptosis. Similar level of apoptosis by miltefosine was noted at 48 h. This clearly indicates that imipramine induces apoptosis in LD parasites much faster than miltefosine. This is in agreement with reports that apoptosis and change in mitochondrial potential are linked phenomenon [35], [36].

LD infection is associated with increase of membrane fluidity [37] as well as defective antigen presentation by APC's [37]. Therefore the antigen presenting ability of imipramine treated MΦs (also defined as APCs) with murine T cells was studied. We observed that successive doses of imipramine restored membrane rigidity, which was in turn coupled with improved antigen presentation ability (Figure 3A & 3B). This may be attributed to the fact that due to the clearance of intracellular parasites upon imipramine treatment, MΦ may regain its normal fluidity. Due to structural similarities to some extent (fused ring structure with side chain), imipramine can mimic cholesterol which acts as a cementing molecule to pack the lipid bilayer [38]. This may be another reason for the restoration of membrane fluidity. Imipramine has already been reported to mimic the action of cholesterol to regulate protein synthesis in SREB (Sterol Regulatory Element Binding) protein synthesis pathway [39].

We have observed that imipramine induces production of leishmanicidal molecules such as superoxide and nitric oxide in MΦ (Figure 4A & 4B). This observation complements other studies that imipramine induced production of TNF-α [12], an important cytokine for antileishmanial defense. The therapeutic role of imipramine in experimental VL infection may be most likely be attributed to a direct leishmanicidal activity both *in vitro* and *in vivo*, its capacity to modulate the host immune response in favour of the host and its ability to induce reactive oxygen species generation on MΦ.

Since imipramine at a dose of 60 µM completely clears the intracellular amastigotes in an *in vitro* macrophage system (Figure 2), we tested the efficiency of imipramine in an *in vivo* model. At a dose of 5 mg/kg/day for 4 weeks, the drug indeed clears >99.5% parasites in hamster infected with either SSG-R or SSG-S LD. We chose to administer oral treatment of imipramine for 4 weeks based on the fact that in humans miltefosine is given orally for 4 weeks [40]. The drug imipramine was found to be equally effective in a murine model where infection was induced by SSG-R parasites (Unpublished observation). The *in vivo* dose of miltefosine was determined based on the animal equivalent of the human dose as described elsewhere [29]. On the other hand, miltefosine treatment for 4 weeks at the dose 17.5 mg/kg/day showed complete clearance of SSG-S but not SSG-R parasites from the spleen and liver of infected hamsters (Figure 5 A–D Group V). The residual SSG-R parasites after miltefosine treatment, even though their number was low, raise concern of cross resistance between miltefosine and SSG. This is perhaps not so surprising since infection with SSG-R parasites upregulates an ABC transporter in the host cells [14] that regulates efflux of both the drugs [17], [41].

Recently there is a great deal of interest for short course of combination treatment regimens [42]. As such, a 10-day treatment protocol has been proposed as an ideal short term regimen by the Drugs for Neglected Diseases Initiative (DNDi) [43]. The DNDi also aims to study new indications for existing medicines in the field of the most neglected diseases. In tune with these recommendations, we also tested the efficacy of imipramine as a monotherapy for a shorter version of treatment, i.e. for 10 days. We again opted to select the dose that provided maximum protection, i.e. 5 mg/kg/day. This regimen showed to clear about 90% of the organ parasites in infected hamsters (Data not shown). Importantly, there is still opportunity to increase the dose of the drug based on the rodent equivalent of human dose [27], also to opt for combination treatment. Imipramine at 5 mg/kg/day for 4 weeks does not affect the hepatic enzymes or creatinine levels in hamsters (Unpublished observation), suggesting that the dose might even be increased further.

Elicitation of effective T cell based host immune response defines the success of antileishmanial chemotherapeutics [44]. Disease severity in experimental animal models infected with SSG-R strains was associated with significantly hampered antigen presentation; antigen-specific T cell activation, low expression of IL-12, TNF-α and IFN-γ, and upregulation of suppressive cytokines IL-10 and TGF-β in murine and hamster models respectively. We therefore tested the immunological parameters associated with successful chemotherapy in SSG-R LD infected animals treated with various therapies.

Enhanced antigen presentation by imipramine treated APCs are also reflected in antigen specific expansion of T cell repertoire in vivo. The skewing of the T cell repertoire towards a Th1 type population is substantiated by the elevated level of IFN γ mRNA expression in splenocytes derived from imipramine treated hamsters.

In vivo treatment with imipramine markedly increased the level of IL-12 in mRNA. The established phase of VL is associated with deactivation of MΦ with severely reduced capacity for production of inflammatory mediators like IL-12 and TNF-α. IL-12 interacts with T cells and induces the initiation and maintenance of Th1 responses via IFN-γ production. IFN-γ and TNF-α are often reported to act synergistically to activate iNOS for the production of NO, the leishmanicidal effector molecule [45]. Strong IL-12 driven IFN-γ coupled with TNF-α triggering by imipramine treatment suggests that these cytokines might be acting in concert to produce NO to effectively kill the parasites.

A growing body of literature correlates IL-10 and TGF-β with susceptibility to Leishmania infection [46]–[54]. Imipramine caused strong suppression of IL-10 and TGF-β production that correlated with successful resolution of infection. Recent reports suggest that resistant parasites modulate the host immunity to exacerbate the ongoing disease pathogenicity [55]. TGF-β is implicated as an important contributor to disease susceptibility or resistance to Leishmania by direct MΦ deactivation [56] and also by increased production of IL-10 [57]. TGF-β being a pleotropic cytokine also suppresses IFN-γ-induced MHC class II expression by inhibiting class II transactivator mRNA [58]. We found significant down regulation of TGF-β mRNA expression in imipramine treated hamsters compared to infected controls. While imipramine treatment attenuates TGF-β expression, it could be causally related to a simultaneous inhibition of IL-10 production [46] with concurrent rescue of MΦ deactivation, thus implying the importance of both the cytokines in disease progression. We like to emphasize the possible role of TGF-β in the outcome of LD infection in hamsters because the IFN-γ/IL-10 ratio changed (1.47 fold) compared to the IFN-γ/TGF-β ratio (1.78 fold) upon imipramine treatment.

The study of the survival kinetics of infected hamsters showed clearly that imipramine treatment increased the life expectancy of the infected hamsters with 90%. In the remainder 10% of the hamsters, death occurred the early time point, the cause of which is not clear. The remaining 90% remained healthy until the termination of the experiment, i.e. 44 weeks post infection. We determined the organ weight (spleen and liver) at 34 weeks and 44 weeks (Table 2) and the parasite burden of the imipramine treated group. Surprisingly, parasites could not be detected in imipramine treated hamsters at the 34^th^ nor at the 44^th^ week and the organ weights were close to normal at 44 weeks post infection. These hamsters at the time of termination of experiment failed to show any parasites but displayed the presence of antileishmanial antibodies, indicating that they were indeed exposed to parasites.

Efficient immune response in the liver depends on the formation of granulomas [59], which is associated with the resolution of hepatic parasite burden [60], [61]. It is well documented that only mature granuloma can develop efficient leishmanicidal mechanism to kill parasites whereas developing immature granulomas lack that efficiency [62], [63]. Parasite killing within the granulomas requires infiltrating monocytes and TNF α [64], although their formation is independent of TNF α family of cytokines [65]. Our previous study with KMP-11 vaccinated hamsters reveals well formed granuloma formation and absence of LD infected Kupffer cells [27]. Our present study showed matured sterile granuloma formation in 4 week of imipramine treated infected hamsters, which was absent in the 12 week infected group (Figure 9) and is associated with the protection. It may be recalled that enhanced maturation of granulomas represents a marker of vaccine induced protection [66].

Orally administered imipramine is rapidly absorbed in the gastro-intestinal tract [9]. Imipramine is a lipophilic compound, binds to albumin, and attains a peak plasma concentration within 2–6 h. This tertiary amine is typically metabolized by demethylation to the secondary and active metabolite, desipramine [9]. Both imipramine and its metabolite desipramine have been found to be equally effective against LD promastigotes [14].

Resistance to SSG is a major problem in the Indian subcontinent and MΦs upregulate both MRP-1 and P-gp upon infection with SSG-R LD leading to efflux of antimonials [15]. Furthermore, circulatory monocytes of kala-azar patients harboring SSG-R LD show over expression of P-gp and MRP-1 [67]. SSG in combination with pharmacological inhibitors of MRP-1 and P-gp favors killing of intracellular SSG-R LD [68]. The tricyclic imipramine is lipophilic and possesses a positive charge due to the nitrogen atom, characteristics that are important to affect the function of P-gp [69] In MDR gene transfected and also in human AML cells ex vivo, such drugs reverse the multidrug resistance phenotype [69]. Thus imipramine will offer an additional advantage since it is a selective P-gp inhibitor. Furthermore, cationic amphiphilic drugs that is basic (pKa 7–8) concentrates on lysosomes [13]. Imipramine being a tertiary amine and weak base will remain as positively charged molecular entity in the body fluids, leading to an affinity towards lysosomes [13], [70]. This unique property is of importance because phagolysosomes constitute the home for intracellular *Leishmania* parasites.

In conclusion, our study clearly indicated that imipramine is more effective than miltefosine and has a strong potential to be considered as an orally active, highly effective, very cheap, affordable chemotherapeutic agent against kala-azar either alone or in combination.

## Supporting Information

Figure S1 **Reduction in splenic parasite burden upon oral miltefosine treatment by serial dilution assay.** Eight-week infected hamsters received miltefosine at a dose of 17.5 mg/kg for 4 weeks and 2 days after last treatment hamsters were sacrificed. The reciprocal of the highest dilution that was positive for parasite growth was considered to be the concentration of parasites per milligram of tissue. Total organ parasite burden was calculated from spleen or liver weight. Results are expressed as % decrease in parasite load with respect to infected control.
(TIF)
