# Supplementary figures and images for: Imipramine Is an Orally Active Drug against Both Antimony Sensitive and Resistant Leishmania donovani Clinical Isolates in Experimental Infection
Source: PLoS Negl Trop Dis. 2012 Dec 27;6(12):e1987. doi: 10.1371/journal.pntd.0001987 (PMC3531496; doi:10.1371/journal.pntd.0001987)

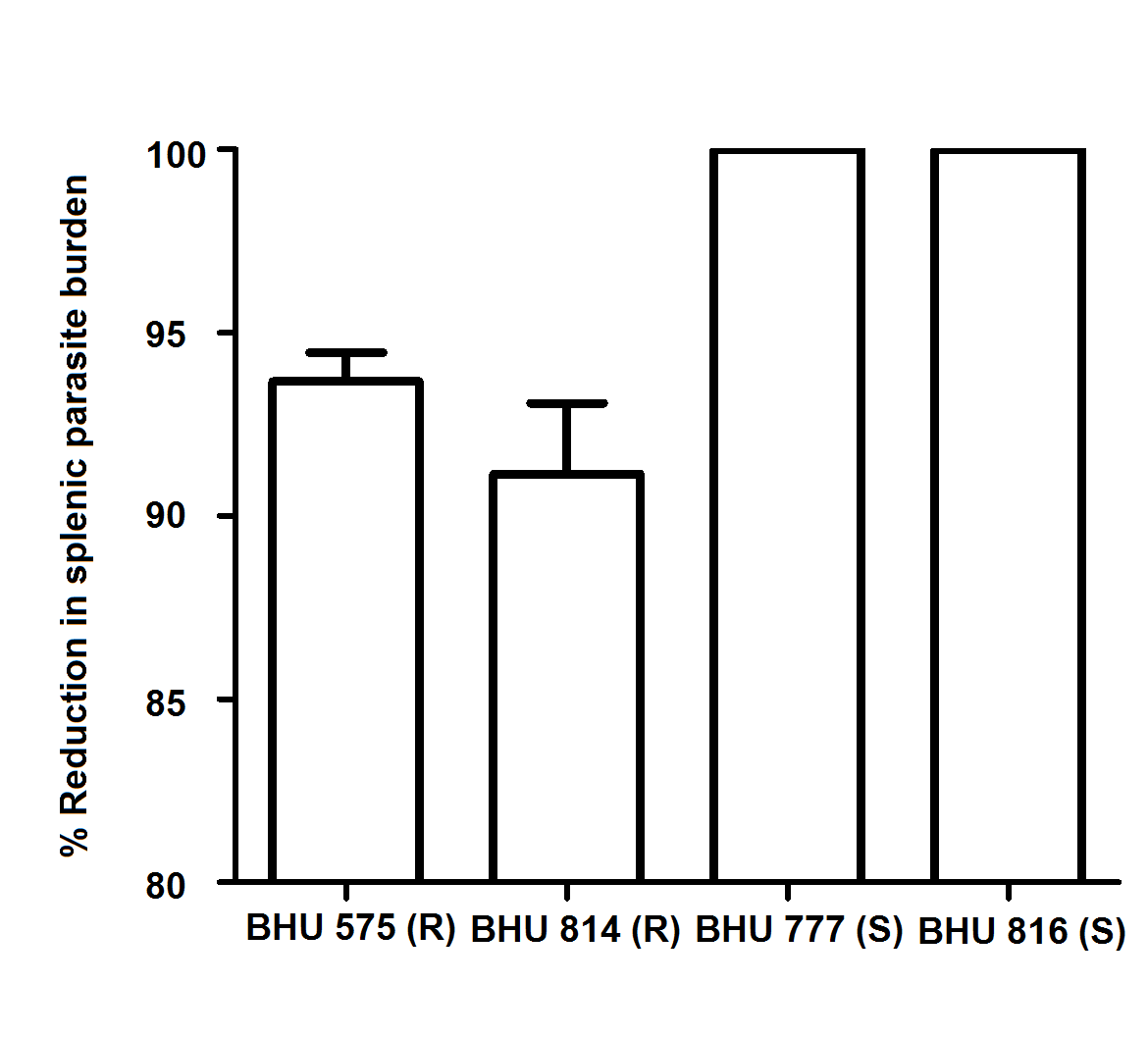

Supplement: Figure S1 — Reduction in splenic parasite burden upon oral miltefosine treatment by serial dilution assay. Eight-week infected hamsters received miltefosine at a dose of 17.5 mg/kg for 4 weeks and 2 days after last treatment hamsters were sacrificed. The reciprocal of the highest dilution that was positive for parasite growth was considered to be the concentration of parasites per milligram of tissue. Total organ parasite burden was calculated from spleen or liver weight. Results are expressed as % decrease in parasite load with respect to infected control. (TIF) [file pntd.0001987.s001.tif]
